# Designing and Implementing the Adaptive Learning Meeting Cycle: The (re)solve Project Experience in Burkina Faso

**DOI:** 10.9745/GHSP-D-22-00217

**Published:** 2023-12-18

**Authors:** Reshma Trasi, Cecelia Angelone, Ginette Hounkanrin

**Affiliations:** a Trasi Duarte Consulting, Alexandria, VA, USA.; b Pathfinder International, Washington, DC, USA.; c Pathfinder International, Ouagadougou, Burkina Faso.

## Abstract

Through the collection and utilization of timely data, program implementers can review feedback and make rapid adjustments and adaptations to interventions while they are in progress.Responsive feedback mechanisms (RFMs), which emphasize flexibility and iterative adaptation, provide opportunities for improving the fit and feasibility of a program, as well as its effectiveness. The (re)solve project—a 5-year, multicountry project testing new, context-specific solutions to address unmet need for family planning—used responsive feedback to ensure products and services designed and implemented were responsive to the context and preferences of health care workers, women, and girls. The adaptive learning meeting (ALM) cycle was designed as an RFM during implementation. This frequent series of rapid, actionable, cross-team meetings builds on several existing frameworks and practices. The ALM cycle used rapid, close to real-time data and observations from a wide range of stakeholders; routine monitoring data; and a structured, facilitated process to examine and act on feedback. Each cycle was repeated every 2 weeks for 3 months in Burkina Faso. During each cycle, the team interpreted data and feedback from multiple sources; assessed pragmatic, actionable options to address the feedback; and identified and agreed upon short- and long-loop adaptations that could improve the implementation process, coordination, efficiency, and outputs of the project. The ALM cycle proved helpful in engendering practices of surfacing and checking assumptions, careful interpretation of data, options analysis, and decision-making. The emphasis was on actionable feedback for improvement of the intervention rather than rigor of results and findings. The (re)solve project's experience with designing, structuring, and implementing the ALM cycle to address challenges and gaps in implementation can be informative for similar programs seeking to implement RFMs in complex and dynamic settings, especially where technology-based RFMs are not an option.

## Abstract (precis)

The (re)solve project's adaptive learning meeting cycle allows programs to adapt while responding to the program implementation context. In Burkina Faso, it also created a team culture that engendered dialogue, problem-solving, and continuous improvement.

## Teaser

**Key Findings:**

- The (re)solve project implemented an innovative project in Burkina Faso in schools to address barriers to contraceptive uptake. The project's iterative design flagged the need for responsive feedback mechanisms (RFMs) at various points. During implementation, the team designed and conducted the adaptive learning meeting (ALM) cycle, a type of RFM customized to suit the needs of the project.
- The ALM cycle followed a 5-step process for group-based learning and problem-solving using monitoring and observational data that allowed a geographically dispersed project team to learn, be agile, and adapt to real-world implementation challenges.
- The ALM cycle resulted in various context-specific adaptations that were responsive to girls, community-based facilitators, and health care workers in Burkina Faso. It had the added benefit of engendering a team culture of problem-solving and continuous improvement.

**Key Implication:**

- Program managers may benefit by learning from the experience of and the steps taken by the (re)solve project in designing and structuring this RFM.

## INTRODUCTION

Responsive feedback mechanisms (RFMs) are not entirely new in international development practice or global health. They build on several approaches such as “participatory monitoring and evaluation,”^1^^,^^2^ “participatory action research,”^3^ “developmental evaluation,”^4^^,^^5^ “problem-driven iterative adaptation,”^6^ and “collaboration, learning and adaptation.”^7^^,^^8^ These approaches all rely on data-informed feedback-decision-action loops, where information is gathered, interpreted, and used by implementers to improve the reach, impact, and effectiveness of programs during and throughout implementation.^9^^,^^10^

Although each of these approaches has distinct, distinguishing elements, they also have common characteristics. They engage and center the voices of the community and local stakeholders who are experts in the context and in identifying solutions through their professional and lived experiences; the practices are flexible, iterative, and repetitive in nature; and they use frequently collected data and dialogue to inform responsive action and identify context-specific program changes and adaptations to improve program effectiveness. Consequently, RFMs provide key opportunities for programs to understand and respond to implementation-related barriers and challenges that emerge while delivering programs in complex social, cultural, economic, and political contexts; use monitoring data; test assumptions from a program theory of change; and make decisions to adapt and improve the implementation approach to maximize results.^5^^,^^11^^–^^14^

An “adapt-as-you-go” approach can also work well in programs where interventions are not predetermined, implementation teams have flexibility to tweak program activities, or innovative services or products are being designed and tested for fit and feasibility in a specific context.^5^^,^^15^ RFMs, when used as part of the broader monitoring and evaluation approach of an innovative pilot program, can serve a purpose that is distinct from summative evaluation models where fidelity, causality, and generalizability are important. These mechanisms can help us understand how an innovative program needs to be fine-tuned and improved to fit into a complex, dynamic system and context. RFMs can be helpful in arriving at a context-specific, feasible model or program that maximizes outcomes. Summative evaluations can help us understand if the model produces the desired outcomes. Where resources are available, RFMs could be combined with summative evaluation approaches, as was the case with the (re)solve project.

RFMs, like the adaptive learning meeting cycle, can help us understand how an innovative program needs to be fine-tuned and improved to fit into a complex, dynamic system and context.

In this article, we describe the (re)solve project and its unique design and the solutions and activities in Burkina Faso. We also describe the project's rapid, actionable, responsive, and virtual RFM—the adaptive learning meeting (ALM) cycle—and discuss its benefits and limitations. The steps we used to design and implement the ALM cycle may be helpful to practitioners in framing and structuring RFMs in similar environments.

## THE (RE)SOLVE PROJECT

**FIGURE 1:**
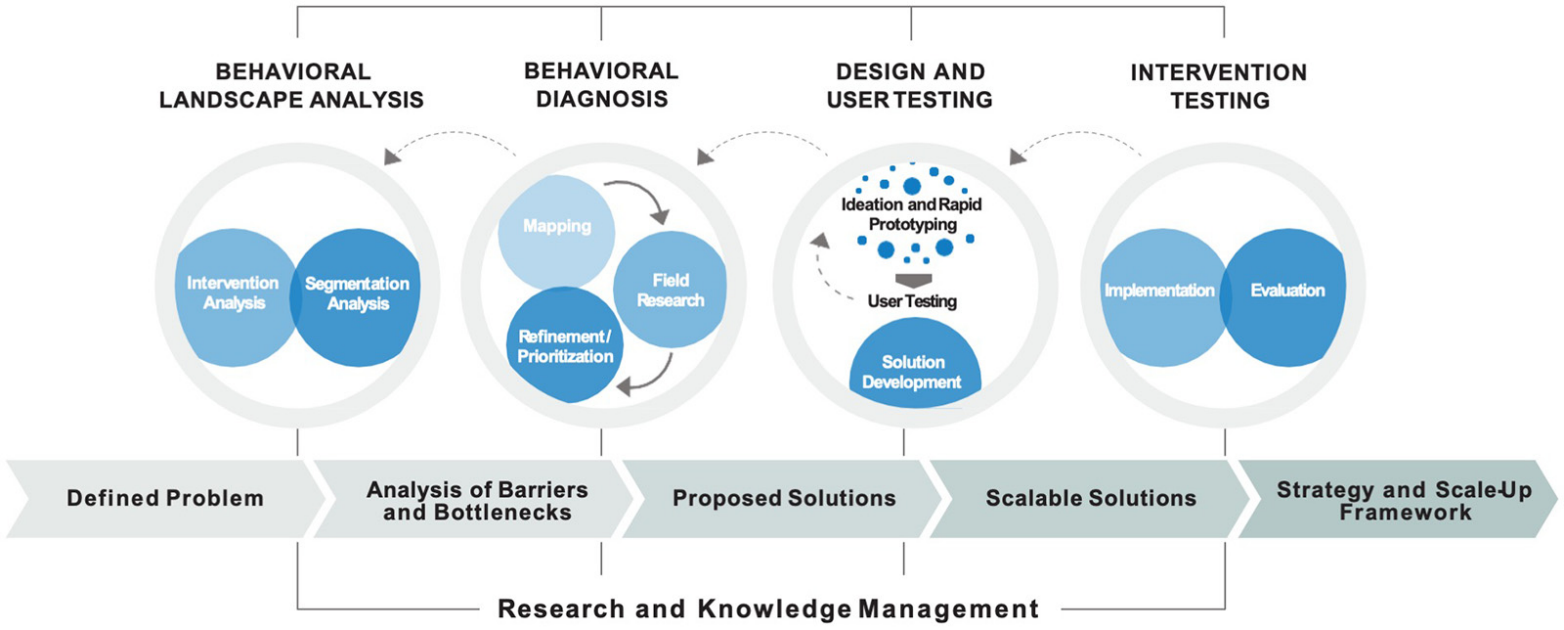
The (re)solve Project Framework

(re)solve was a 5.5-year (2016–2022), 3-country project that combined consumer insights, behavioral economics, human-centered design, and public health to discover what stops women in Bangladesh, Burkina Faso, and Ethiopia from using modern contraceptive methods (referring to all hormonal methods, emergency contraception, and condoms) even when they express a desire to avoid pregnancy. The project used an iterative, phased process (Figure 1) that sought to surface context-specific insights about contraceptive decision-making using sequential segmentation analysis and behavioral diagnosis. These analytical and diagnostic methods draw on multiple social and behavioral theories, such as the Integrated Behavior Model^16^ and neurocognitive biases (e.g., anchoring effect, zero-risk bias, and spotlight effect).^17^ We used these insights to design, test, and evaluate adaptable, scalable, user-responsive solutions that addressed unmet need for family planning.

At the (re)solve project's heart was the conviction that one size does not fit all, even within a demographically similar subpopulation, such as unmarried adolescents and youth. The (re)solve project used segmentation analysis and behavioral economics to identify barriers and insights that prevent married garment workers in urban Bangladesh, postpartum women in rural Ethiopia, and unmarried school-going adolescents in urban Burkina Faso from taking up a contraceptive method when they want to prevent an unintended pregnancy. We then designed and tested unique behavioral solutions for each subpopulation that addressed their context-specific barriers. These solutions were either new products (a digital counseling training app, a board game, and an integrated voice-response system) or new job aids (planning tools and a pregnancy risk assessment tool). These data-informed behavioral solutions aimed to address the needs, motivations, and lived experiences of women and girls.

We designed feedback mechanisms and iterative adaptation into the project framework at 2 key points: (1) user testing during the design and user testing phase and (2) ALM cycle during the intervention testing phase.

During the design and user testing phase, we gathered feedback and reactions to early prototypes (i.e., low-fidelity versions of the solutions) from the implementation team, technical advisory groups, health care workers, and women and girls. We discarded or improved subsequent prototypes based on feedback provided primarily by women, girls, and health care workers. We consider user testing as an example of an RFM, where stakeholders provide detailed feedback on a solution prototype. These data informed iterations and improvements made to subsequent versions. This process was repeated over several weeks with different users, including women, girls, teachers, health care workers, and community health workers, until we arrived at solutions that were acceptable, easily understood, and easy to use by the intended users of the solutions.

Once we developed the solutions and advanced them to the intervention testing phase with a larger population, we developed a theory of change, which informed a detailed monitoring, evaluation, and learning plan and tools for each country. During the intervention testing phase, we used the theory of change as the “compass” for project implementation, for identification of assumptions, metrics for routine monitoring, and as the basis for a summative evaluation of the intervention. Although the summative evaluation findings were designed to inform recommendations for future replication and scale-up, we needed another feedback mechanism that would test our implementation-related assumptions and address any implementation-related issues. The mechanism needed to tap into routine monitoring data, project team observations and experiences, and capture lessons and adaptations.

This second feedback mechanism that was implemented during intervention testing was dubbed the ALM cycle (described in detail later). Unlike the user testing phase that aimed to assess the acceptability and usability of the prototypes, the ALM cycle was created specifically to improve the fit and feasibility of the respective interventions in each country's context. Unfortunately, due to several contextual factors, including the onset of COVID-19 in early 2020, we were not able to proceed to intervention testing in Bangladesh. A regional conflict and communication blackouts prevented us from using the cycle during intervention testing in Ethiopia. Therefore, this article focuses on using the ALM cycle in Burkina Faso.

The ALM cycle was created specifically to improve the fit and feasibility of the respective interventions in each country's context.

### Examining Behavioral Barriers to Contraceptive Uptake in Burkina Faso

In Burkina Faso, the behavioral landscape analysis and behavioral diagnosis phases focused on exploring and examining the behavioral barriers that adolescent schoolgirls aged 14–18 years were uniquely facing in voluntarily taking up a modern contraceptive method if they were sexually active and wanted to avoid getting pregnant. The adolescent fertility rate is high at 98 per 1,000 women aged 15–19 years.^18^ Fifty-seven percent of pregnancies among this age group are unintended.^19^ The Ministry of Health recognizes the importance of reducing teenage pregnancy and unsafe abortions in Burkina Faso.^20^ Behavioral barriers that emerged through our work included: girls reported a lack of control or voice in health decisions; they perceived that providers were biased toward unmarried girls seeking modern contraceptives in health centers; unmarried girls did not explicitly consider the consequences of unprotected sex; unmarried girls did not use contraceptives because they had other options to avoid pregnancy such as the calendar method; girls did not use contraceptives because the risk of infertility, no matter how small, was perceived as too great; and girls did not go to the health facility because there were no cues to do so (unless they became pregnant or were ill).^21^

The (re)solve project was managed and implemented by a 4-person team in Burkina Faso, led by a project manager, a program officer, and 2 part-time site supervisors. Together, they facilitated the training and engagement of facilitators from community-based organizations with which the project worked. The team worked directly with school administrators on scheduling and with health facilities to collect routine service delivery data. Throughout the project and especially implementation, the country team was supported by 2 part-time and 2 full-time U.S.-based colleagues who supported all 3 countries with program management, iterative design, cross-program learning, and monitoring and evaluation. More information on the roles and responsibilities of those contributing to the (re)solve project's implementation in Burkina Faso is included in the Table.

**TABLE.** (re)solve Project Implementation Roles and Responsibilities in Burkina Faso

	Description	Roles and Responsibilities
Community-based facilitators	Individuals recruited by 2 community-based organizations (1 in Ouagadougou and 1 in Bobo-Dioulasso) and trained by the Burkina Faso team on the project and intervention	Implemented the intervention in schools by facilitating the board game, distributing the passports, and answering girls' questions.Collected data on gameplay and reported observations and feedback to Burkina Faso team.
Health facility staff	Health facility providers and reception staff oriented to the project and intervention by the Burkina Faso team	Implemented the solution set in health facilities by displaying posters, wearing name badges, and offering youth-friendly services to schoolgirls with passports. Collected data on passport use and reported feedback to Burkina Faso team.
Implementation team	Burkina Faso-based program manager, program officer, and 2 part-time site supervisors	Led implementation of the intervention in Burkina Faso and engaged with ministries and relevant stakeholders, including school administrations and health facilities. Trained CBOs and community-based facilitators and oriented health providers. Participated in all steps of the ALM cycle and led decision-making for implementation adaptations.
Global project team	U.S.-based project director, program officer, and part-time monitoring, evaluation, and learning advisor from Pathfinder International	Collated data, facilitated the ALM, and provided guidance based on the project's mandate and constraints.
Evaluation team	U.S.-based senior social and behavioral scientist/evaluation lead and research and program associate from consortium partner	Designed evaluation data collection instruments, conducted baseline and endline evaluation, analyzed data, and reported findings.

Abbreviation: ALM, adaptive learning meeting.

It is also important to note the context in which the (re)solve project was implemented in Burkina Faso, which added to the complexity of this project. The project was designed to be “embedded” within a larger at-scale project, Yam Yankre (My Choice), delivering sexual and reproductive health (SRH) services to married and unmarried women of reproductive age. We had to ensure that anything we designed could then be implemented by the Yam Yankre project and by civil society organizations in Burkina Faso. The (re)solve project benefited by being embedded in a larger project and leveraged their regular health facility supervision visits to gather monitoring data that were useful to our work. We sought guidance and approval from the project's advisory board during each phase and kept them closely informed. We implemented the interventions in 2 geographically distant and diverse urban areas, Ouagadougou and Bobo-Dioulasso. Because we were implementing in schools and health facilities, we engaged with both the Ministry of Health and the Ministry of Education, which required navigating and aligning their respective recommendations and approvals. In addition, our team faced teacher strikes, ongoing unrest and security risks, and the onset of the COVID-19 pandemic.

### The Burkina Faso Interventions

**FIGURE 2:**
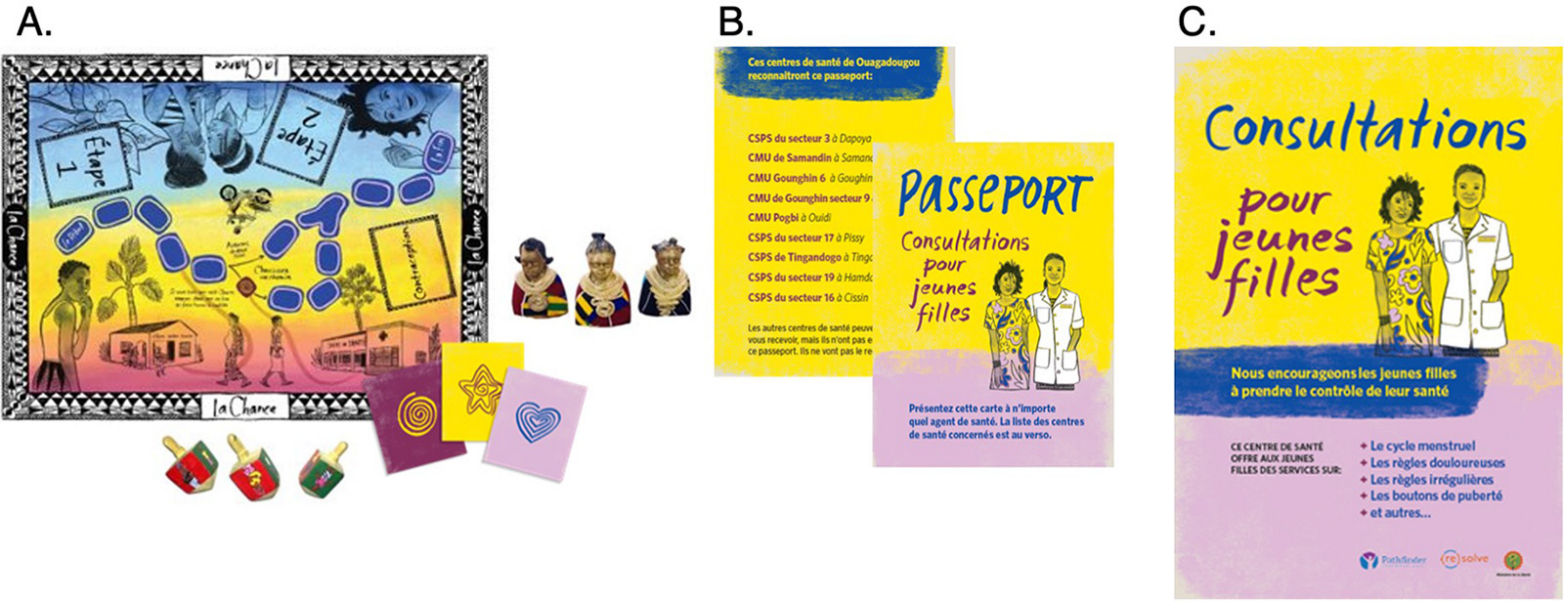
The (re)solve Project Intervention Components in Burkina Faso: (A) La Chance Board Game and Playing Pieces; (B) Health Passport (front and back); (C) Poster

Through the design and user testing phase, the (re)solve project generated a mutually reinforcing set of the following 3 connected interventions or “solutions” (Figure 2).^22^

#### La Chance Board Game

Through an interactive board game, schoolgirls explored a series of real-life scenarios informed by the behavioral landscape analysis and behavioral diagnosis. The game was played at school in teams, and a trained community-based facilitator reinforced key messages and answered questions at the end. We designed it to correct myths and misconceptions about specific methods through trivia-based learning and group dialogue; elevate a player's pregnancy risk perception by providing opportunities to experience this risk through real-life scenarios; help her explore personal, relational, and social trade-offs associated with an unintended pregnancy; and explore strategies on how to seek health care discreetly.

#### The Health Passport

After they played the game, each girl received a multi-use “passport” for herself and 2 copies to share with friends of her choosing. The passport cued her to follow through on her intention to avoid pregnancy by visiting a participating health facility for SRH information and services.

#### Poster and Nametag

Posters at participating health facilities publicized available noncontraceptive, adolescence-related, primary health care services for girls (such as menstrual cramps and puberty-related skin changes) and aimed to normalize their presence at the facility, especially if they were seen or recognized by a family or community member. The health care workers' name tags provided visual confirmation to girls who went to a participating facility that the health care worker was trained in delivering SRH information and services to unmarried adolescent girls. Health care workers received a half-day orientation on the provision of youth-friendly health services.

During the intervention testing phase, from December 2019 to March 2020, the (re)solve project implemented the solutions in 16 randomly selected secondary schools, 8 each in Bobo-Dioulasso (Bobo) and Ouagadougou (Ouaga). We conducted orientation sessions on the solutions, their rationale, and the implementation plan with school principals and parent-teacher associations of intervention and control schools, as well as with parents of girls in participating schools. Thirty-two community-based facilitators implemented the board game and distributed passports to girls across the 16 participating schools. Two facilitators were assigned to each intervention school, and they conducted 1–2 games per day. A total of 3,120 girls in grades 4ème (grade 9) and 3ème (grade 10) played La Chance between December 2019 and March 2020. Facilitators distributed 11,908 passports to girls in this time frame.

Due to the iterative nature of the project, we had the rare dual opportunity and mandate to adapt as well as evaluate the intervention. The (re)solve project conducted a randomized controlled trial with 16 intervention and 16 control schools.^21^ The results showed that the solutions improved girls' beliefs and attitudes and significantly increased the number of girls who went to the health facilities for SRH information or services.^20^ The ALM cycle allowed us to adapt and improve implementation.

## APPLYING THE ALM CYCLE TO IMPROVE IMPLEMENTATION

The implementation and evaluation plan in Burkina Faso had time constraints because of the school year and required coordination between multiple stakeholders such as schoolgirls, teachers, school administrators, facilitators, and health care providers. Because the game was being implemented in schools with girls in grades 9 and 10, the implementation team had to sensitize parents to the game, complete a baseline survey of over 2,000 girls, train community-based facilitators to facilitate the game, train health care workers on youth-friendly services and orient them to the solutions, implement the game, and administer an endline survey all within the school year (i.e., from October 2019 to April 2020) and before the start of national grade 10 exams. The game was played during lunch breaks and “free periods,” when girls would be available, making the timeline even more constricted. Implementation was planned within a tight, 4-month timeline (December 2019–March 2020) so as not to disrupt exam preparation schedules while still providing time for the endline survey to be administered. To be able to determine if the solutions worked well within a tight timeline and had the potential to be scaled up, we needed to monitor, observe, learn, and adjust in this real-world setting in as close to real-time as possible. Our geographically dispersed team needed a mechanism that would allow us to rapidly process the data and information generated through our monitoring and learning tools and inform adaptations necessary to improve the program. In addition to centering the voices of women and girls, using regular monitoring data, and being iterative, we needed a mechanism with the following characteristics.

Our geographically dispersed team needed a mechanism that would allow us to rapidly process the data and information generated through our monitoring and learning tools and inform adaptations necessary to improve the program.

- **Rapid:** We were implementing within a tight timeline, so the mechanism had to be rapid while keeping the project timeline on track.
- **Internally inclusive:** We needed our implementation and evaluation teams around the table to ensure we were interpreting the data correctly, based on the local context, and discussing the implication of any adaptations on the theory of change and evaluation design.
- **Action-based:** We needed to collectively identify adaptations and make decisions quickly.
- **Virtual:** We needed a semistructured and consistent format for the mechanism so we could use our geographically dispersed team's time efficiently.

These characteristics shaped the purpose, design, and structure of the ALM cycle, which allowed us to collectively use and interpret data to identify adaptations to improve the implementation process, coordination, efficiency, outputs, and outcomes of the project.

**FIGURE 3:**
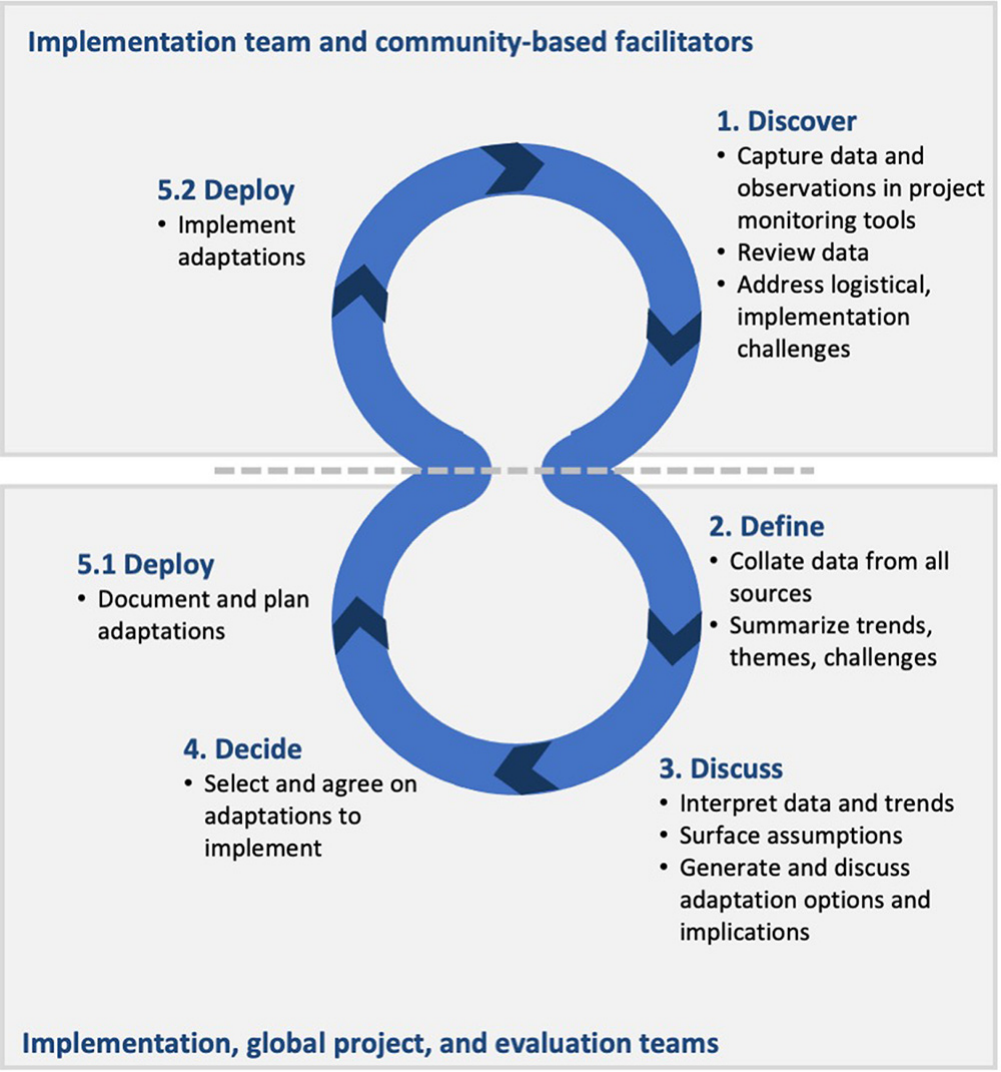
The 5 Steps of the (re)solve Adaptive Learning Meeting Cycle

We designed a fit-for-purpose feedback mechanism, which we called the ALM cycle. In each cycle, routine monitoring data were collected, reviewed, collated, and organized. Each cycle culminated in a group meeting, where decisions about implementation adaptations were made and recorded. Each ALM cycle consisted of the “5Ds”: discover, define, discuss, decide, deploy (Figure 3). These 5 steps are adapted from participatory approaches such as “problem-driven iterative adaptation,” “developmental evaluation,” appreciative inquiry,^23^ and iterative processes involved in user-centered software product design.^24^ The first 2 steps took place in Burkina Faso with the implementation team and community-based facilitators; the subsequent 3 steps describe the structure and flow of the virtual, fortnightly meeting with the global team.

### Discover

This step consisted of data collection from various data sources to understand if the solutions were accepted and used by girls and health care workers, understand how the solutions were being implemented, and identify challenges being faced by the implementation team. We designed 3 routine monitoring and reporting tools: (1) weekly monitoring reports from community-based facilitators, (2) school and health facility site visit tools, and (3) reflection diaries from the implementation team.

The community-based facilitators completed weekly monitoring reports consisting of a few key metrics that could signal implementation challenges: the number of girls who played the game in Bobo and Ouaga, the number of girls who refused to play the game and their reasons for refusal, and the number of passports distributed. At the end of each week, the implementation team conducted on-site, in-person, weekly group meetings with the community-based facilitators in both Ouaga and Bobo to review these monitoring reports and respond to questions or challenges faced while facilitating the games. These weekly sessions served as opportunities for cross-site knowledge sharing, local problem-solving, logistical coordination improvement, and peer-to-peer learning among facilitators. The implementation team addressed and solved most coordination and implementation-related problems at these meetings. For example, facilitators shared that girls going to health facilities early in the implementation period reported that they did not have a positive experience despite the half-day youth-friendly services training conducted for all health care providers. Based on this feedback, during regular, on-site monitoring visits to health facilities, the implementation team reinforced messaging around the importance of providing youth-friendly services. In another example, facilitators shared that schoolgirls wanted to know if they had to pay out-of-pocket for counseling services or contraceptives at the health facilities. The implementation team printed and shared a list of contraceptives that were available for free and the out-of-pocket cost for others and made this available to all facilitators to share with girls.

The implementation team also conducted fortnightly on-site follow-up visits to participating health facilities and schools and recorded their observations using site visit tools. The health facility visits were used to gather routine data in the site visit tools on the number of girls who were coming to the health facilities with passports and to determine if the health facility staff were wearing the name badges provided by the project. The school visits were useful in gathering feedback from administrators and teachers on the intervention and addressing any logistical or administrative concerns they might have. The site visit tools gave guidance on what to look for during the visits, such as checking that posters were on display in the schools and facilities, and provided space for capturing notes on discussions with school administrators or facility health providers.

Once collected, the implementation team entered this feedback and any on-site data and observations from the school and health facility visits into reflection diaries. By capturing the thoughts, considerations, and suggestions of the implementation team after their site visits, the diaries ensured that the feedback from the school and health facility-based stakeholders was included and raised during the ALM cycle, along with challenges experienced by the implementation team and the community-based facilitators.

### Define

The implementation team shared the collated and aggregated routine monitoring data and reflection diaries, as well as the notes, solutions, and decision points from the weekly meetings with the U.S.-based (re)solve program officer. The program officer then collated and reviewed the monitoring data trends and notes, identified common themes and observations, highlighted questions raised by the implementation team for the global project team, and shared them electronically with the implementation and global project teams a few days before the fortnightly team meeting. These themes and observations, along with the challenges, defined the agenda for these meetings. Every member of the team reviewed the data, notes, and questions.

### Discuss, Decide, and Deploy

Every 2 weeks for 3 months, the implementation team, global project team, and U.S.-based evaluation team attended a meeting conducted virtually over Microsoft Teams. The Burkina Faso implementation team included the program manager, program officer, and 2 part-time site supervisors. The U.S.-based global project team included the project director, program officer, and monitoring, evaluation, and learning lead. We maintained detailed meeting notes for each meeting. Each meeting usually lasted 90 minutes and followed a facilitated semistructured format. The first part of the meeting was dedicated to surfacing assumptions and raising questions on or concerns around the data trends and observations to ensure that our interpretations aligned with the data from the monitoring reports and the observations of the implementation team. The implementation team's inference of the data took precedence, given their proximity to the implementation and their deep knowledge of the real-world settings in which we were implementing the program.

The next part focused on addressing unsolved challenges raised by the facilitators or issues the implementation team wanted to elevate and on deciding the course correction. Together, the implementation and global project teams made collective decisions on which adaptations to implement. This usually consisted of generating options, discussing the pros and cons of each option, and deciding on an adaptation to implement. During the discussions, team members raised concerns if any proposed adaptation would not work in the local context; potentially reduce the effectiveness of the intervention; alter the theory of change; contaminate the evaluation design; introduce unintended consequences, especially physical, psychological, or social harm to the schoolgirls; or place an unsustainable burden on the implementation team, the health system, or the education system. For example, there was a high demand from girls to play the game multiple times, and in Bobo, several girls played the game twice before the implementation team learned about this from the community-based facilitators. The project did not have the resources or time to uniformly play the game twice across all intervention schools. Additionally, this would have had significant implications on our evaluation sample size to test for diverse levels of exposure within the evaluation design. After discussing the implications, the implementation and global project teams agreed to proceed with the original implementation plan of girls playing the game once.

If an adaptation was accepted, it was then deployed, meaning implementation steps were identified and responsibilities were agreed upon and assigned. We documented the selected adaptation and any relevant strategies to mitigate the risk of unintended consequences and revisited them at the subsequent fortnightly meeting to determine if they were being implemented as planned and whether they were having the desired impact on the challenges they were intended to address.

## RESULTS

The ALM cycle allowed a geographically dispersed project team to learn, be agile, adapt, and solve problems while using monitoring data, experiential knowledge, and project documentation as a cornerstone for adaptation. Using the ALM cycle facilitated the identification of what we called “short-loop” and “long-loop adaptations.”

### Short-Loop Adaptations

Short-loop adaptations were organic or deliberate changes to the intended implementation of the solution and related activities that could be implemented within the scope and timeline of the project. For example, some facilitators struggled with SRH knowledge and terminology and with providing the information girls were requesting after playing the game despite receiving preliminary training from the implementation team. To address this, the implementation team started reserving half of every weekly meeting with the facilitators for training and knowledge reinforcement on requested SRH topics. Because the weekly topics were directly responsive to the questions raised by the facilitators, they differed between Bobo and Ouaga. Another example of a short-loop adaptation came about when the implementation team learned some girls were bringing their health passports to nonparticipating health facilities. Although we could not provide youth-friendly service provision training to these additional facilities mid-implementation the implementation team visited the identified facilities and explained the purpose of the passport and the project to ensure that girls would not be turned away and could be referred to participating facilities.

### Long-Loop Adaptations

In contrast, long-loop adaptations were recommendations from school administrators, facilitators, or schoolgirls that could not be implemented within the scope and timeline of the project but ought to be considered for future replication and scale-up efforts. Many of these recommendations were included in the final evaluation report and in national dissemination workshops. One such example was the sustained interest from girls, facilitators, and school administrators in expanding the game to younger grades, boys, and out-of-school girls. Some girls also wanted to play the game with their boyfriends. Many girls also had basic questions about anatomy and reproduction, as well as more specific questions about cervical cancer and sexually transmitted infections. To address these other interests, adaptations would need to be made to the game and tested with different audiences. The original cards were designed based on the (re)solve project's behavioral diagnosis findings, but new cards could be added to address girls' common questions and content could be adjusted for different age levels. More behavioral diagnosis will also be needed to adapt the game's real-life scenarios to the lived experiences of boys.

## DISCUSSION

The ALM cycle had several benefits. First, acknowledging the contextual expertise of the implementation team and the community-based facilitators at the beginning put them into problem-solving mode. Contextual knowledge and lived experience are areas of expertise, and we deferred to our Burkina Faso team to make decisions related to their context. Having clearly identified expertise and roles ensured that we were tapping into the collective expertise and experience of our team across geographies. As a result, many implementation challenges were solved pragmatically and creatively at the weekly meetings with facilitators. A future area of inquiry could explore whether processes such as these can shift perceptions of ownership, power, and hierarchy within geographically dispersed teams. Second, centering the voices of schoolgirls and facilitators when considering the project's adaptation choices ensured that the project was responsive, primarily to schoolgirls, facilitators, and the context in which the interventions were being implemented and intended to be scaled. Third, through its structure, the ALM cycle avoided a skewed focus on analyzing the problem, which could have resulted in a blame game or “analysis paralysis.” Rather, the ALM cycle shifted the emphasis to action, learning, and adaptation. This shift allowed the implementation and global project teams to collectively recognize when something was not working, learn from it, and move quickly to find a solution. The focus on dialogue, discussion, and problem-solving also meant that less time, if any, was spent questioning the rigor and quality of the data or identifying the need for more data. We used the best data available to us captured through our monitoring tools to make and act on decisions quickly. Over time, team members came to meetings with options identified, and our problem-solving approach was further accelerated. Fourth, the ALM cycle imbued a culture of continuous improvement and efficiency across the team. For example, at each meeting, close attention was paid to which data were being used and which were not. Based on this, some metrics and reflection questions were removed to keep data collection and documentation manageable.

The ALM cycle shifted the emphasis to action, learning, and adaptation. This shift allowed the implementation and global project teams to collectively recognize when something was not working, learn from it, and move quickly to find a solution.

Finally, the team habits and team culture engendered by the ALM cycle had ramifications beyond its intended design and use in Burkina Faso. Although this was neither an outcome we were tracking nor one we expected, we include it here so that future implementers or researchers may consider tracking or examining this in the future. The 5Ds helped immensely when Bobo-Dioulasso and Ouagadougou went into COVID-19 pandemic lockdown, schools closed in April 2020, and the future seemed uncertain. The planned endline survey, in-depth interviews, and key informant interviews required in-person data collection in schools and health facilities. The implementation team brought real-time information about curfews and school closures to the meetings, along with an analysis of which aspects of the program could continue and which ones could not. The evaluation team quickly brainstormed ideas, presented options, and discussed the pros, cons, implications, and feasibility of each option given the constraints. We collectively decided on and successfully completed a phone-based endline survey, which included data collectors interviewing girls on the phone about sexual activity while using creative ways to ensure maximum safety and confidentiality for girls who were taking these calls from their respective homes during the COVID-19 lockdown.^25^

We observed that the ALM cycle benefited our teams in Ethiopia and Bangladesh, as well. These team-based solution-oriented practices helped us rapidly gather contextual information and data, generate options, and decide on an alternate evaluation design when COVID-19 lockdowns forced the (re)solve project to abandon a longitudinal randomized controlled trial planned in Ethiopia. Additionally, we used the 5Ds to monitor the rapidly evolving pandemic in Dhaka, Bangladesh, the health system response, and garment factory closures over a period of several months. The options we generated to continue the project, which consisted of providing a combined digital and in-person solution to garment factory workers through pharmacies and urban health clinics, would have meant placing our Bangladesh team, our service delivery partners, and garment factory workers at additional COVID-19 infection risk. The painful decision to eventually phase out the program in Bangladesh emerged from our collective team discussions. The global project team also undertook a similar process to present several future scenarios to the donor when COVID-19-related uncertainties affected our timeline and results in Bangladesh and Ethiopia.

Because of the multiple benefits, we believe that the ALM cycle and similar feedback mechanisms like user testing, where action is taken based on feedback from project implementers, women, and girls, are a good investment of time and resources. Although it took time to collect and collate the data, we observed that the process became faster and easier with each cycle. The implementation team spent half a day per week facilitating the group meeting with the facilitators. They spent less than half a day entering data into the monitoring tools. Every fortnight, the U.S.-based program officer spent less than a day collecting, collating, identifying themes, and developing an agenda for discussion. The rest of the global project team and evaluation team spent less than half a day every fortnight reviewing the data and themes and participating in the meetings. The ALM cycle could be feasible to implement in large, at-scale service delivery projects. It may not require a fortnightly cadence, which was appropriate for the piloting of innovative services requiring rapid iteration and adaptation. A well-structured and facilitated ALM-like cycle could be conducted on a quarterly or semiannual basis depending on the frequency of monitoring data collection.

### Limitations

Other opportunities to be inclusive and participatory, as well as to improve cross-learning and follow-up within and between RFMs, were missed. We struggled to design a feedback mechanism between schoolgirls and facilitators during implementation, and schoolgirls were not included in the weekly meetings. Implementation demands on the facilitators, school attendance during the week for the schoolgirls, time zone differences, and technology challenges prevented inclusion of the facilitators and schoolgirls at the fortnightly meetings. Feedback from girls would also have required additional approvals from the ethical review committee, causing delays in our tight implementation and evaluation timeline. If ethical approval can be sought early on, including girls and facilitators in the ALM cycle could be feasible. However, implementers may need to balance the value of the rich feedback they may receive with the money and time needed to generate and use the feedback to improve the intervention. Despite this limitation, a few short-loop adaptations were identified by schoolgirls and facilitators and shared with the implementation team. For example, the girls selected a “class monitor” in each grade to remind girls who were scheduled to play the game on any given day that it was their turn. This simple shift helped facilitators reduce the time for game setup and completion. In another example, schoolgirls decided to go to health facilities in groups for mutual support rather than risk going alone and being seen by a family member or relative.

## CONCLUSION

In closing, we recognize that RFMs and program adaptations are not “once and done,” especially when programs are being implemented in evolving or uncertain contexts because the context can change, sometimes significantly. These mechanisms require careful planning, dedicated time and resources, and careful facilitation of frequent discussions. However, when implemented and nurtured over time, RFMs, like the (re)solve project's ALM cycle, offer a structure and format that can center and elevate the feedback and lived experience of women, girls, and health care workers; engender a commitment to adapt programs to be responsive to this feedback; and build a strong team culture that imbues agile adaptation and problem-solving at all levels. When implemented in the spirit of continuous improvement and centering the voices of in-country implementers and community stakeholders, RFMs can allow program teams to recognize what is not working, learn from this experience, and adapt to maximize outcome and impact. It could be argued that, despite being somewhat labor-intensive to thoughtfully design, plan, implement, and facilitate, RFMs may prove cost-effective in the long run by preventing the investment of finite resources in programs that are neither responsive to nor maximize outcomes for the communities they serve.

## Notes

**Peer Reviewed**

**First published online:** November 3, 2023.

## References

[1] Jacobs A, Barnett C, Ponsford R. Three approaches to monitoring: feedback systems, participatory monitoring and evaluation and logical frameworks. IDS Bulletin. 2010; 41(6): 36–44. Accessed October 18, 2023. https://bulletin.ids.ac.uk/index.php/idsbo/article/view/1887

[2] Guijit I. *Participatory Approaches. Methodological Briefs: Impact Evaluation 5*. UNICEF; 2014. Accessed October 18, 2023. https://www.betterevaluation.org/sites/default/files/Participatory_Approaches_ENG.pdf

[3] Chevalier JM, Buckles DJ. *Handbook for Participatory Action Research, Planning and Evaluation*. SAS2 Dialogue; 2021. Accessed October 18, 2023. https://7ffe3e06-6b70-4b9a-859d-686a6a37b74d.filesusr.com/ugd/11f418_1706d6db86ca44c09e2b2a727e3587e7.pdf

[4] Patton M. *Developmental Evaluation: Applying Complexity Concepts to Enhance Innovation and Use*. Guildford Press; 2010.

[5] Developmental evaluation. Better Evaluation. Accessed October 18, 2023. https://www.betterevaluation.org/en/plan/approach/developmental_evaluation

[6] Andrews M. Pritchett L, Woolcock M. Escaping capability traps through Problem-Driven Iterative Adaptation (PDIA). World Dev. 2013;51:234–244. Accessed October 18, 2023. 10.1016/j.worlddev.2013.05.011

[7] U.S. Agency for International Development (USAID). *Evidence Base for Collaborating, Learning, and Adapting: Summary of the Literature Review*. USAID; 2020. Accessed October 18, 2023. https://usaidlearninglab.org/sites/default/files/resource/files/cla_literature_review_update_march_2020_final.pdf

[8] Anderson MB, Brown D, Jean I. *Time to Listen: Hearing People on the Receiving End of International Aid*. CDA Collaborative Learning Projects; 2012. Accessed October 18, 2023. https://www.cdacollaborative.org/wp-content/uploads/2016/01/Time-to-Listen-Hearing-People-on-the-Receiving-End-of-International-Aid.pdf

[9] U.S Agency for International Development (USAID). *Rapid Feedback Monitoring, Evaluation, Research and Learning (Rapid Feedback MERL)*. USAID; 2019. Accessed October 18, 2023. https://www.usaid.gov/sites/default/files/2022-05/RAPID_Factsheets_2019.pdf

[10] Whittle D. *How Feedback Loops Can Improve Aid (and Maybe Governance)*. Center for Global Development; 2013. Accessed October 18, 2023. https://www.cgdev.org/sites/default/files/WhittleFeedbackessay_1.pdf

[11] The Curve. *Evidence for Decision-Making: Incorporating Evidence Into a Responsive Feedback Approach*. The Curve; 2021. Accessed October 18, 2023. https://the-curve.org/wp-content/uploads/2020/04/201112_The-Curve-Evidence-for-Decision-Making_Final.pdf

[12] CDA Collaborative Learning Projects. Feedback Mechanisms in International Assistance Organizations. *CDA Desk Study*. CDA Collaborative Learning Projects; 2011. Accessed October 18, 2023. https://www.alnap.org/help-library/feedback-mechanisms-in-international-assistance-organizations-cda-desk-study

[13] The Curve. *Success Stories: Inspiring Case Studies of Continuous Improvement*. The Curve; 2021. Accessed October 18, 2023. https://the-curve.org/wp-content/uploads/2020/05/Case-Studies-Success-Stories.pdf

[14] Viswanath K, Synowiec C, Agha S. Responsive feedback: towards a new paradigm to enhance intervention effectiveness. Gates Open Res. 2019; 3:781. 10.12688/gatesopenres.12937.2. 31131370 PMC6480401

[15] International Rescue Committee (IRC); Mercy Corps. *Adapting Aid: Lessons From Six Case Studies*. IRC/Mercy Corps; 2016. Accessed October 18, 2023. https://www.rescue.org/sites/default/files/document/701/adaptingaidreportwithcasestudies.pdf

[16] Montano DE, Kasprzyk D. Theory of reasoned action, theory of planned behavior, and the Integrated Behavioral Model. In: Glanz K, Rimer BK, Viswanath K, eds. *Health Behavior and Health Education: Theory, Research, and Practice*. 4th ed. Josey-Bass; 2008.

[17] Cognitive biases: a list of the most relevant biases in behavioral economics. The Decision Lab. Accessed October 18, 2023. https://thedecisionlab.com/biases

[18] Adolescent fertility rate (births per 1,000 women ages 15–19)– Burkina Faso. The World Bank. Accessed October 18, 2023. https://data.worldbank.org/indicator/SP.ADO.TFRT?locations=BF

[19] The Alan Guttmacher Institute. *Adolescents in Burkina Faso: Sexual and Reproductive Health*. The Alan Guttmacher Institute; 2004. Accessed October 18, 2023. https://www.guttmacher.org/sites/default/files/report_pdf/rib3-04.pdf

[20] Burkina Faso. Ministere de la Sante. *Plan National de Developpement Sanitaire 2011–2020*. Ministere de la Sante; 2011. Accessed October 18, 2023. https://www.childrenandaids.org/sites/default/files/2018-05/Burkina_Faso_Nat_Health_Strategy_2011-2020%20Fr.pdf

[21] (re)solve Project. *Shifting Young Girls' Sexual and Reproductive Health Attitudes, Beliefs, Norms, and Intentions in Burkina Faso: Evaluation Results From the (re)solve Project*. Pathfinder International; 2020. Accessed October 18, 2023. https://www.pathfinder.org/wp-content/uploads/2023/01/resolve-Burkina-Faso-Technical-Brief_ENG_r11.pdf

[22] Hinson L, Schaub E, Pliakas T, et al. A Game, a Passport, and a Poster: Changing Contraceptive Attitudes, Intentions, and Behaviors Among School Girls in Urban Burkina Faso. *(re)solve Project Evaluation Report*. Pathfinder International; 2020. Accessed October 18, 2023. https://www.pathfinder.org/wp-content/uploads/2023/01/resolve-Burkina-Faso-Evaluation-Report_r7.pdf

[23] 5-D cycle of appreciative inquiry. AI Commons. Accessed October 18, 2023. https://aicommons.champlain.edu/learn/appreciative-inquiry-introduction/5-d-cycle-appreciative-inquiry/

[24] Illés A, Funtek F. Product design process: steps to designing a product people will love. uxstudio. May 3, 2021. Accessed October 18, 2023. https://uxstudioteam.com/ux-blog/product-design-process-steps/

[25] Hinson L, Schaub E, Aya N, Tamboura-Diawara A, Trasi R. Pivoting in the face of the unknown: five adaptations for effective phone-based data collection during COVID-19. International Center for Research on Women. September 2, 2020. Accessed October 18, 2023. https://www.icrw.org/pivoting-in-the-face-of-the-unknown-five-adaptations-for-effective-phone-based-data-collection-during-covid-19/

